# Resuscitation Promoting Factor (Rpf) from *Tomitella biformata* AHU 1821^T^ Promotes Growth and Resuscitates Non-Dividing Cells

**DOI:** 10.1264/jsme2.ME12122

**Published:** 2012-10-26

**Authors:** Indun Dewi Puspita, Moe Uehara, Taiki Katayama, Yoshitomo Kikuchi, Wataru Kitagawa, Yoichi Kamagata, Kozo Asano, Cindy H. Nakatsu, Michiko Tanaka

**Affiliations:** 1 Graduate School of Agriculture, Hokkaido University, N9 W9, Kita-ku, Sapporo, Hokkaido 060–8589, Japan; 2 Institute for Geo-Resources and Environment, National Institute of Advanced Industrial Science and Technology (AIST), Tsukuba, Ibaraki 305–8566, Japan; 3 Bioproduction Research Institute, National Institute of Advanced Industrial Science and Technology (AIST), 2–17 Tsukisamu-Higashi, Toyohira-ku, Sapporo, Hokkaido 062–8517, Japan; 4 Department of Agronomy, Purdue University, West Lafayette, Indiana 47907, USA

**Keywords:** *Tomitella biformata*, resuscitation promoting factor, non-dividing cells, permafrost ice wedge

## Abstract

Functional variation of Rpf, a growth factor found exclusively in *Actinobacteria*, is differentiated by its source and amino acid sequences. Only purified Rpf proteins from three species have been studied so far. To seek new Rpfs for use in future studies to understand their role in *Actinobacteria*, the objective of this study was to identify *rpf* gene homologs in *Tomitella biformata* AHU 1821^T^, a novel *Actinobacteria* isolated from permafrost ice wedge. Amplification using degenerate primers targeting the essential Rpf domain led to the discovery of a new *rpf* gene in *T. biformata*. Gene structure and the deduced Rpf domain amino acid sequence indicated that this *rpf* gene was not identical to previously studied Rpf. Phylogenetic analysis placed *T. biformata* Rpf in a monophyletic branch in the RpfB subfamily. The deduced amino acid sequence was 44.9% identical to RpfB in *Mycobacterium tuberculosis*, the closest functionally tested Rpf. The gene was cloned and expressed in *Escherichia coli*; the recombinant Rpf protein (rRpf) promoted the growth of dividing cells and resuscitated non-dividing cells of *T. biformata*. Compared to other studies, this Rpf was required at higher concentrations to promote its growth and to resuscitate itself from a non-dividing state. The resuscitation function was likely due to the highly conserved Rpf domain. This study provides evidence that a genetically unique but functional Rpf can be found in novel members of *Actinobacteria* and can lead to a better understanding of bacterial cytokines in this phylum.

The resuscitation promoting factor (Rpf) is a protein that has been shown to promote the growth of active cells and to resuscitate non-dividing cells (17). Thus far, purified Rpf proteins from only three species, *Micrococcus luteus* (17), *Mycobacterium* (*Myc.*) *tuberculosis* (19), and *Corynebacterium glutamicum* (8), have been studied, although the activity of spent media from a broader range of bacteria carrying *rpf* genes has been reported (28). Putative and functionally tested *rpf* genes in different bacteria vary in length, genetic structure, and in host specificity and activity; however, all sequences include a conserved domain (Rpf domain) with lysozyme-like activity that is approximately 70 amino acids long (25). The exact mechanism involving Rpf in the resuscitation of non-dividing cells is not known but likely involves its capacity to cleave cell wall components, producing peptidoglycan fragments that can served as signaling molecules for growth initiation (10, 16). Rpf produced by a bacterium not only affects its own growth but also has cross species activity (17, 19, 29).

Bioinformatics analysis has found homologs of Rpf only in the high G+C Gram-positive bacterial phylum (25). All these sequences have an Rpf domain but differ in the presence of other domains and their number. For example, the RpfB subfamily is composed of a signal peptide, three copies of the DUF348 domain, a G5 domain and Rpf domain. Eight subfamilies of *Actinobacteria* Rpf have been proposed and two other families with distantly related proteins in Firmicutes have been putatively identified. The functions of eight Rpf proteins have been studied: Rpf from *M. luteus* from the LysM subfamily; five Rpfs from *Myc. tuberculosis* that are in Rpf subfamilies A, B, C, D, and E; *C. glutamicum* Rpf2 from the RpfB subfamily, and *C. glutamicum* Rpf1 from the Corynebacterium subfamily. Now with the availability of over 2,100 completed bacterial genome sequences (>3,000 including draft genomes), a BLASTp search using the Rpf domain sequence indicated that it is exclusively found within the phylum *Actinobacteria* (Table S1). Of the 306 publically available draft or completed *Actinobacteria* genome sequences, 202 genomes representing 29 families have one to seven copies of deduced Rpf amino acid sequences. The prevalence and number of Rpf in *Actinobacteria* genomes suggests that it plays an important role in this group. The growth and resuscitation function of Rpf may be fundamental in the life cycle of some non-spore-forming bacteria, which include most genera in the *Actinobacteria*, for which little is known about the mechanisms controlling dormancy and re-growth.

Bacteria with Rpf sequences are not limited to specific environments; they have been isolated from diverse locations including the human microbiome, plant rhizosphere, contaminated soils, deep sea marine sediment, hot spring runoff, and sewage sludge (Table S1). Current understanding of the roles played by Rpf, especially in respect to the diverse environments in which *Actinobacteria* are found, is still limited. In addition to the fundamental knowledge gained about bacterial cytokines and intercellular signaling systems in bacteria, two important functions of Rpf drive current studies. First, of medical importance is the role Rpf plays in the reactivation of non-dividing *Myc. tuberculosis* cells (9, 17) from the latent state of the disease (32). Secondly, in environmental microbiology, the recognition that only a fraction of cells in most environments are readily cultivated has led to the proposal of a number of strategies to improve cultivation efficiency (24), including the use of Rpf to cultivate *Actinobacteria* (28). Along this line it is necessary to obtain different Rpf homologs to cultivate the diversity of yet uncultivated populations of *Actinobacteria*, many with potentially beneficial biotechnological attributes.

Despite the conservation of the Rpf domain there is variation in activity and cross species recognition of Rpf tested from different bacterial sources and with different sequences. For example, a comparison of the laboratory strain of *M. luteus* and strains isolated from amber found differences in lysozyme resistance that were attributed to differences in the linker region length of their *rpf* genes (12); therefore, we hypothesized that new Rpf would be found in novel *Actinobacteria* isolated from unique environments. The objective of this study was to determine the presence and activity of an *rpf* homolog in *Tomitella biformata* AHU 1821^T^ (=DSM 45403^T^ = NBRC 106253^T^), a novel member of the suborder Corynebacterineae isolated from a permafrost ice wedge in Fox Tunnel, Alaska (11). The phylogenetic distance from other known function Rpf will provide a measure of the uniqueness of *T. biformata* Rpf. The biological functions of the gene product in both growth promotion and in the resuscitation of non-dividing cells of *T. biformata* were tested.

## Materials and Methods

### Organisms and culture conditions

*Tomitella biformata* AHU 1821^T^ (=DSM 45403^T^ =NBRC 106253^T^) was aerobically grown at 20°C, the optimum growth temperature for this strain (11), with shaking (140 rpm) in Bacto Tryptic Soy Broth without dextrose (BD, USA) supplemented with 2% (w/v) fructose (TSBF), on TSBF plates solidified with 2% (w/v) agar or in defined minimal medium (30) that was slightly modified (mMMF) by increasing the concentration of phosphorus and nitrogen by adding 17.2 mM K_2_HPO_4_ and 37.4 mM NH_4_Cl and using 27.7 mM fructose as the carbon source. All experiments were started with cultures that had been grown to the mid-logarithmic growth phase and stored in glycerol solution (20% v/v) at −80°C. Glycerol stock culture (2% v/v) was first grown in TSBF medium until the mid-log phase and then transferred (1% v/v) to an appropriate growth medium. *Escherichia coli* TOP 10 (Invitrogen, USA) used for cloning and expression was grown in Luria-Bertani (LB) medium supplemented with 50 μg mL^−1^ ampicillin (LB-amp) (Nacalai Tesque, Japan) or kanamycin (Wako, Japan) where appropriate.

### Determination of the *rpf* gene sequence

Chromosomal DNA of *T. biformata* was extracted and purified following a previously described method (22). A putative *rpf* gene fragment from *T. biformata* was obtained by PCR of purified DNA (50 ng) using degenerate primers 2RpfF (5′-AYCAACACSGGHAACGG-3′) and 2RpfR (5′-CCABGCKCCCCARCCCTG-3′) conserved in the Rpf domain of *M. luteus* Rpf protein (GenBank accession no. Z96935 positions 181–333). Touchdown PCR amplification was used (5 min at 95°C; then 11 cycles of 1 min at 95°C, 1 min at 60°C (decrease 1°C per cycle), and 1 min at 72°C; followed by 20 cycles of 1 min at 95°C, 1 min at 51°C, and 1 min at 72°C; and finally 10 min at 72°C). The amplicons were purified using the SUPREC-PCR kit (Takara, Japan), inserted into the pGEM-T Easy vector system (Promega, USA), transformed, and sequenced using the ABI Prism Big Dye Terminator Cycle Sequencing Ready Reaction Kit (Applied Biosystems, USA) following the manufacturers’ protocols. The sequence of the amplicon was analyzed using BLASTx (1) to identify the best matching protein sequence. The amplified product was labeled and hybridized to a Southern blot made from *Hind* III-digested *T. biformata* genomic DNA. An approximately 1.9-kbp *Hind* III fragment hybridized with the probe was self-ligated to obtain a circular DNA product that was used as the template (10–50 ng) for inverse PCR using KOD Plus Ver. 2 (TOYOBO, Japan). Two primers InvJ2F1 (5′-GCAGATCGAGAT TGCCGAGAAG-3′) and InvJ2R1 (5′-GGTGCTGGGGGAGAA CTGCA-3′), which were constructed within the probe sequence, were used to obtain the entire ORF of the putative *rpf* gene. The thermal cycler conditions used for inverse PCR were 5 min at 95°C; then 10 cycles of 10 s at 98°C, 30 s at 63°C (decrease 0.5°C per cycle), and 2 min at 68°C; followed by 20 cycles of 10 sec at 98°C, 30 sec at 58°C, and 2 min at 68°C; and finally 5 min at 68°C. The purified products (approximately 1,800 bp) were inserted into the pCR-Blunt II-TOPO cloning vector (Life Technologies, USA) and sequenced. Sequence assembly and analysis were carried out using Genetyx (GENETYX Corporation, Japan). Potential signal peptide and conserved domains within the putative *rpf* gene were determined using the Signal P 3.0 server (6) and CD-Search programs (14), respectively.

### RNA extraction and reverse transcriptase-PCR

Cultures of *T. biformata* growing in mMMF were collected throughout the exponential growth phase, after 48, 72, 96, and 144 h of incubation, and immediately mixed with 2 volumes of RNAprotect Bacteria Reagent (Qiagen, USA) and stored at −80°C until RNA was extracted. RNA was extracted using the RNeasy Mini Kit (Qiagen) after cells had been digested using lysozyme (Wako) and Proteinase K (Qiagen) and mechanically disrupted in Lysing Matrix B (MP Biomedicals, Solon, OH USA) using a Multi-beads Shocker (Yasui Kikai, Japan). Reverse transcription of template RNA (20 ng) was performed using *T. biformata rpf* gene specific primers (J2TF2, 5′-CGCCGTCGTCGTCGGATTCCA-3′ and J2TR2, 5′-ACGCGGAGGCCAAGCTTGCTG-3′) and the OneStep RT-PCR Kit (Qiagen) following the manufacturer’s protocol. Absence of DNA contamination was confirmed by omitting the reverse transcription step according to the manufacturer’s protocol.

### Expression and purification of rRpf protein

Recombinant Rpf (rRpf) was produced using the pBAD/gIIIB expression vector (Invitrogen) and purified as previously described for *M. luteus* (20), except that LB was used to grow *E. coli* TOP 10 instead of SOB. The *rpf* homolog without its native N-terminal secretory peptide was amplified from *T. biformata* genomic DNA using KOD Plus with two primers pBADgIIIb XhoIF (5′-CCG**CTC GAG**CGCCGTCGTCGTCGG-3′) and pBADgIIIb XbaIR2 (5′-GC **TCTAGA**ACGCGGAGGCCAAGCTTGCTG-3′) (restriction sites are in bold) using the following thermal cycler program: 5 cycles of 10 s at 98°C, 30 s at 70°C (decrease 0.5°C per cycle), and 72 s at 69°C; followed by 25 cycles of 10 s at 98°C, 30 s at 67°C, and 72 s at 69°C; and finally 7 min at 68°C. The restriction enzymes, *Xho* I and *Xba* I, were used to digest the ends of the PCR product for insertion into the corresponding sites in the pBADgIIIB vector. After the sequence was verified, the pBAD/gIIIB plasmid carrying the *rpf* homolog with a hexa-histidine tag was transformed into *E. coli* TOP 10.

The *E. coli* clone was grown in LB-amp medium and expression of the *rpf* gene was induced by addition of 0.01% (w/v) L-arabinose (Wako, Japan). Recombinant *E. coli* cells without L-arabinose induction were processed in parallel to obtain a recombinant protein-negative fraction for activity analysis. Cells were lysed following the manufacturer’s protocol. Supernatants were collected and His-tagged recombinant Rpf protein was recovered by nickel affinity chromatography (HisTrap HP column, GE, UK) and separated on an AKTA Explorer 100 system (GE) using a 20 to 400 mM imidazole gradient at 4°C according to the manufacturer’s protocol.

The expressed proteins were detected by SDS-polyacrylamide gel electrophoresis (PAGE) and Western blot hybridization using the anti-His antibody. Protein fragments from the SDS-PAGE gel transferred by electroblotting to a PVDF membrane (ProBlott; Applied Biosystems), following the manufacturer’s instructions, was used for N-terminal sequence determination. Amino acid sequence analysis was performed on a Procise 492 HT (Applied Biosystems) in the Instrumental Analysis Division, Equipment Management Center, Hokkaido University, Japan. Freshly prepared protein solutions (used on the same day or stored at 4°C for use the next day) were desalted and separated from low molecular weight molecules by centrifugation through an Amicon Ultra-4 filter unit (Millipore, Ireland), diluted and sterilized by passage through a 0.22-μm PVDF membrane (Millipore). Protein concentration was determined using the Quick Start Bradford Reagent kit (Bio-Rad, USA) with spectrophotometric detection (DU 640; Beckman, USA) following the manufacturer’s protocol. The approximate protein concentration used in experiments was estimated based on the deduced molecular weight of the putative Rpf from *T. biformata* (38,000 g/mol).

### Preparation, enumeration and observation of non-dividing cells

Non-dividing cells were induced by incubation under oxygen-limited conditions. Inoculum prepared from a glycerol stock pre-culture was transferred into 20-mL mMMF in 30-mL tubes (in triplicate) that were tightly capped with butyl rubber stoppers (Maruemu, Japan) and crimped with a 20-mm aluminum seal cap (Systech, Japan) then incubated at 20°C with shaking (140 rpm) for 10 d followed by 50 d without shaking. Numbers of culturable cells were determined by plating cultures onto TSBF agar in triplicate and incubating at 20°C for 7 d. Live and membrane-damaged cells from the liquid cultures were discriminated by staining with the Live/Dead Baclight Bacterial Viability kit (Invitrogen) following the manufacturer’s instructions modified by using a SYTO 9:PI proportion of 1:2. Stained cells were counted in a Neubauer counting chamber (Hirschmann EM Techcolor, Germany) using an epifluorescence microscope (BX50; Olympus, Japan). Fluorescent signal from at least 10 fields was captured by CCD camera (VB-7010; Keyence, Japan) to count more than 1,000 total cells. The estimated percentage of non-dividing cells was calculated using the formula:

$$Non\;dividing\;cells\;(\%)=\frac{number\;of\;live\;cells-number\;of\;culturable\;cells}{number\;of\;live\;cells}\times 100$$

Differences in morphological characteristics of cells were determined by TEM analysis of cultures from (i) non-dividing cells (60 d of oxygen limitation in mMMF), (ii) early logarithmic growth (TSBF for 42 h), and (iii) stationary growth (TSBF for 72 h) phases. Cultures were harvested and prefixed in glutaraldehyde (2.5% v/v in 0.1 M sodium phosphate buffer, pH 7.2) at 4°C overnight, followed by fixation in 1% (w/v) osmium tetroxide at 4°C for 60 min. The fixed cells were suspended in 0.5% (w/v) aqueous uranyl acetate for 2 h at room temperature and embedded in 1.5% (w/v) agarose prior to dehydration using an ethanol gradient series. The dehydrated blocks were embedded in Epon 812 resin. Ultrathin sections were cut using an ultramicrotome (Ultracut-N; Leichert-Nissei, Japan), mounted on copper grids, and stained with uranyl acetate and lead citrate. Sections were imaged using a transmission electron microscope (80 kV; H-7600; Hitachi, Japan).

### Determination of rRpf protein activity

The biological activity of rRpf protein was determined using low inoculum concentrations of cells in the logarithmic phase and non-dividing cells of *T. biformata*. Cells in the logarithmic phase were grown in 10-mL TSBF at 20°C, harvested, washed twice with mMMF, and resuspended in the same volume of mMMF medium. Serially diluted cell suspensions (100 μL) were transferred to 10-mL mMMF containing rRpf fractions at final concentrations of 0.25 or 5 μg mL^−1^. For non-dividing cell assays, bacteria from oxygen-limited cultures were harvested, washed, and serially diluted to eliminate the remaining dividing cells from suspensions used for resuscitation experiments. Diluted cell suspensions were transferred to 5-mL mMMF medium containing the rRpf fraction at a final concentration of 2 or 20 μg mL^−1^. Triplicate cultures were grown with shaking (140 rpm) in test tubes fitted with silicon stoppers. Cell growth was monitored daily by OD_600_ measurements using a spectrophotometer (Spectronic 20D+; Thermo Scientific, USA). Sterilized distilled water (SDW) and eluted non-induced cell lysate were used as negative controls.

### Phylogenetic analysis of putative Rpf sequences

Phylogenetic analysis was performed using the deduced amino acid sequence from the *T. biformata* putative *rpf* gene sequence and all known and putative Rpf proteins from all other species with completed publically available genome sequences obtained from the Integrated Microbial Genome (IMG) (15) database. In cases in which multiple genomes of the same species were sequenced, only one was used for sequence alignment (Table S1). Amino acid sequences were aligned using ClustalW2 software (13) and the default setting for the Gonnet series for protein weight matrix. The robustness of neighbor-joining tree topology was assessed by bootstrap analyses (7) based on 1,000 replications.

### Nucleotide sequence accession number

The nucleotide sequence for the entire *rpf* gene from *T. biformata* was deposited in GenBank under accession number AB723682.

## Results and Discussion

### Characterization of an *rpf* homolog in *T. biformata*

A 162-bp fragment initially amplified from genomic DNA of *T. biformata* using degenerate primers 2RpfF and 2RpfR had a deduced amino acid sequence with 74% similarity to Rpf protein in *M. luteus*. Using the PCR fragment as a probe, it hybridized to an ~1.9 kb *Hind* III digested fragment of *T. biformata* genomic DNA. The nucleotide sequence of this 1,865-bp fragment included a putative 1,119 bp ORF. BLASTn analysis indicated that the highest sequence match was to *Rhodococcus opacus* B4 (762/1057 bp, 72%), in which Rpf has not been studied. No other sequences provided a match over the full length of the gene. A short region (900–1,000 bp) matched the transglycosylase domain of some other *Actinobacteria*. BLASTp analysis indicated that the highest amino acid sequence match, 58% identity and 73% similarity, was to RpfB from *Rhodococcus erythropolis* strain SK121 (GenBank accession ZP_04388698) and strain PR4 (GenBank accession YP_002767809). Messenger RNA corresponding to the *rpf* gene homolog was detected by RT-PCR throughout the exponential growth phase of *T. biformata* cells, indicating that this gene was being expressed in growing cultures.

**Fig. 1:**
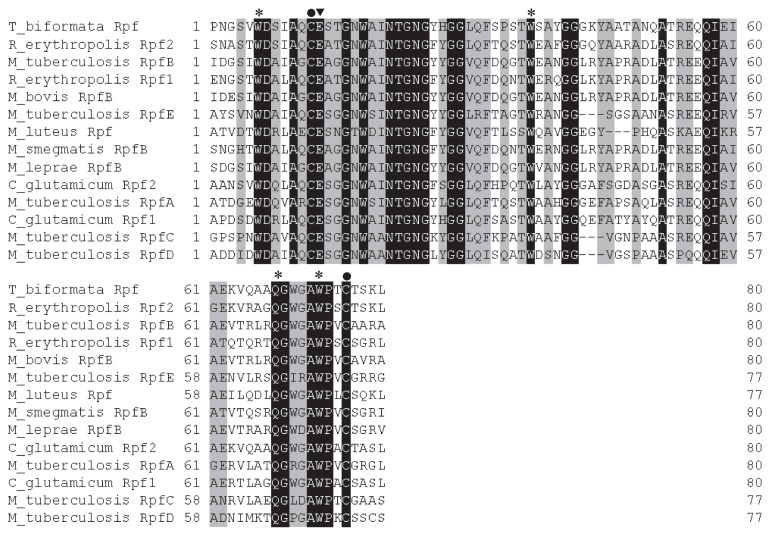
Alignment of deduced amino acid sequences of the Rpf domain from *Tomitella biformata* (AB_723682), *Rhodococcus erythropolis* Rpf2 (YP_002767809), *Rhodococcus erythropolis* Rpf1 (YP_002767808), *Corynebacterium glutamicum* Rpf2 (NP_600137), *Mycobacterium tuberculosis* RpfB (NP_215525), *Mycobacterium bovis* RpfB (NP_854693), *Mycobacterium leprae* MLBr (YP_002502930), *Mycobacterium smegmatis* RpfB (YP_889678), *C. glutamicum* Rpf1 (NP_600048), *Myc. tuberculosis* RpfA (NP_215382), *Myc. tuberculosis* RpfC (NP_216400), *Myc. tuberculosis* RpfD (NP_216905), *Myc. tuberculosis* RpfE (NP_216966), and *Micrococcus luteus* (YP_002957485) using GENETYX software. Identical residues are depicted against a black background and similar residues are in gray. Conserved amino acid residues correspond to the muralytic activity sites (▼), oligosaccharide binding site (*), and cysteines predicted to form a disulphide bridge (●) within the Rpf domain (3, 4, 20).

The deduced amino acid sequence of the putative *rpf* ORF (372 aa) included a 30 amino acid signal peptide, two copies of the DUF348 (domain of unknown function) domain in the N-terminal region, a G5 domain with putative cell-wall adhesive function (26) and an Rpf domain at the C-terminal end (3). The genetic arrangement differed from the functionally studied RpfB in *Myc. tuberculosis*, which has three copies of the DUF348 domain and a shorter linker region between the second DUF348 domain and the G5 domain (25). It also differed from Rpf2, studied in *C. glutamicum*, which also has two copies of DUF348 but a much longer linker region between the two DUF348 copies and a shorter linker region between the second DUF348 domain and the G5 domain (25). Only one of these domains has been studied in detail, and the Rpf domain has been shown to be essential for resuscitation of non-dividing cells of *Actinobacteria* (3, 4). The specific amino acid residues involved in muralytic activity in *M. luteus* Rpf (20), oligosaccharide binding in *Myc. tuberculosis* RpfB (3, 4) and disulphide bond formation (20) were conserved in the *T. biformata* Rpf domain (Fig. 1). Conservation of this region in Rpf from *T. biformata* led us to speculate that it can also cleave peptidoglycan. The functions of DUF348 and the G5 domain are not known but there is an obvious need to determine their role in Rpf; however, there is evidence that differences in the length of linker regions can modify function. *M. luteus* strains carrying Rpf with different linker lengths differed in lysozyme resistance and association of Rpf with the cell wall (12). Further research is needed to understand the function and relationship between the various domains and their linker regions.

### Phylogenetic analysis

**Fig. 2:**
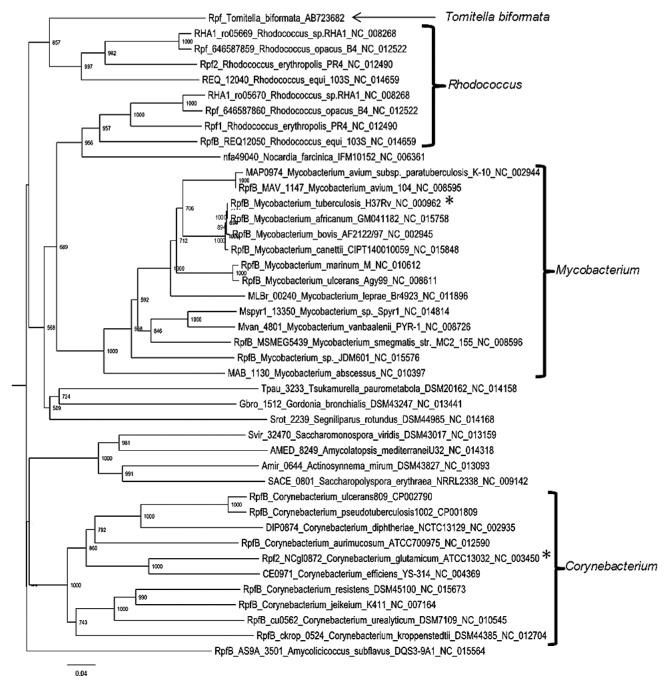
Neighbor-joining tree of Clustal W2 alignment of the deduced amino acid sequence from *T. biformata rpf* gene and sequences of putative members of the RpfB sub-family from completed genome sequences (full tree in Fig. S1). Bootstrap analysis of 1,000 reiterations with values >50% shown at nodes. Asterisks distinguish proteins whose functional activities have been determined.

Phylogenetic analysis using the deduced Rpf amino acid sequence from *T. biformata* and Rpf found in all completed genome sequences placed it within the RpfB subfamily (25) (Fig. S1). Examination of the RpfB subfamily (Fig. 2) revealed that *T. biformata* branched separately and was distant from the functionally tested RpfB from *Myc. tuberculosis* (46% identity, 61% similarity, GenBank accession NP_215525) and Rpf2 from *C. glutamicum* (43% identity, 59% similarity, GenBank accession NP_600137). The tree also indicated that RpfB sequences from the same genera tended to branch together. Outside of the conserved amino acids in the Rpf domain the sequences varied among different Rpf sequences (Fig. 1) and the phylogenetic tree of just this region indicated that the sequence from *T. biformata* is more closely related to the Rpf1 domain in *C. glutamicum* than to RpfB from *Myc. tuberculosis* (Fig. S2). This may be an important observation since Rpf from the different subfamilies of *Mycobacterium* have been shown to be active in different concentration ranges. For example, a broader concentration range of RpfB (1.4–137 pM) from *Myc. tuberculosis* is active in resuscitating non-dividing cells of *Myc. bovis* compared to RpfA (1.6 pM) (19).

### Expression and purification of the rRpf protein

**Fig. 3:**
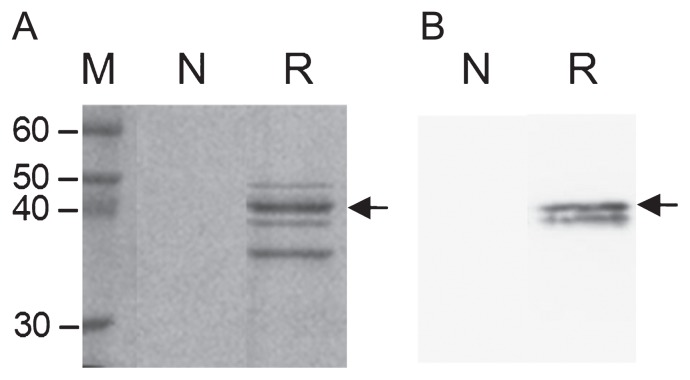
Expression and purification of histidine-tagged recombinant Rpf (rRpf) protein confirmed by (A) SDS-PAGE and (B) Western blot hybridization analysis using anti-His antibody. Arrow shows that 42-kDa protein had the first 13 amino acid residues of the expected calculated size (38 KDa) of rRpf protein: M: molecular weight standard, N: non-induced *E. coli* cells lysate fraction, R: histidine-tagged recombinant Rpf protein fraction.

Production of rRpf by the *E. coli* clone resulted in substantial lysis of the cells, previously described to be related to Rpf accumulation (20) (data not shown). This lysis was not observed in non-induced *E. coli* cultures. After purification, rRpf protein product produced four bands on an SDS-PAGE gel, estimated to be 32, 39, 42 and 49 kDa. No bands were seen in lanes of purified cell lysate from non-induced recombinant cells that were used as negative controls (Fig. 3). Only two of the bands, 39 and 42 kDa, produced strong signals on a Western blot when hybridized with an anti-His antibody. Both these bands were larger than the calculated expected size of 38 kDa but overestimation of Rpf size on SDS-PAGE gels has been previously reported (20, 31). The two protein variants that did not hybridize with the anti-His antibody may have undergone further proteolysis during purification (resulting in a smaller protein) or may have been an immature form of rRpf protein. rRpf of *M. luteus* was expressed using the same vector and also produced several forms of rRpf; using zymogram analysis they found that these multiple protein variants were all active (20).

Sequence analysis of the N-terminal end of the four bands from the SDS-PAGE gel indicated that only three bands had protein sequences that matched the deduced Rpf from *T. biformata*. Only the first 12 amino acid residues of 42-kDa protein matched positions 31–42 of Rpf, the predicted cleavage site of the *rpf*-gene product from *T. biformata*. Two variants had N-terminal amino acid residues matching other regions of Rpf; 39-kDa protein corresponded to positions 53–67 of Rpf and 32-kDa protein to positions 113–127. Truncated forms of *M. luteus* Rpf and *Myc. tuberculosis* RpfB have been reported to be more stable and retain muralytic activity (5, 18, 20); therefore, it was difficult to know if these variants were active. The 15 N-terminal region of 49 kDa protein corresponded to the geneIII signal sequence from the expression vector. This mixture of rRpf proteins was used for growth promotion and resuscitation experiments but it was difficult to estimate the proportion of biologically active molecules among the eluted proteins. The detection of these various protein variants suggests that the *E. coli* system used in this study was not ideal for the expression of this *T. biformata* protein and future studies should consider the use of a genetic system from a more closely related genus such as *Rhodococcus* (23).

### Growth-promoting activity of rRpf fraction

**Fig. 4:**
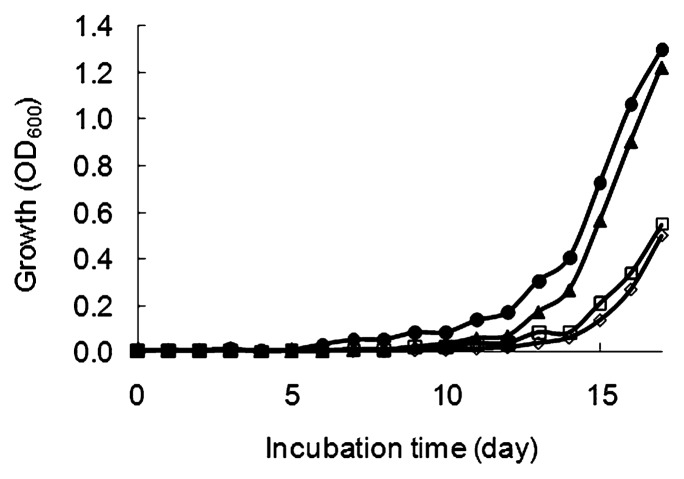
Growth-promoting activity of rRpf on *Tomitella biformata* culture. Logarithmic phase *T. biformata* cells grown in TSBF medium were washed, properly diluted and transferred to mMMF medium with addition of rRpf fraction to the final concentration of 5 μg mL^−1^ (●) and 0.25 μg mL^−1^ (▲). Culture with sterilized distilled water (⋄) and the non-induced cell lysate fraction (□) served as a control. Initial cell concentration of *T. biformata* in mMMF culture was 6.76×10^2^ CFU mL^−1^. Two independent experiments were conducted and one representative data set is shown in this figure.

The addition of the rRpf fraction promoted the growth of *T. biformata* dividing cells in a low initial cell concentration (6.76×10^2^ CFU mL^−1^) in mMMF medium. The apparent lag phase was shortened from 12 d to 10 and 5 d in the culture amended with rRpf of 0.25 and 5 μg mL^−1^, respectively (Fig. 4).

### Non-dividing *T. biformata* cells

**Fig. 5:**
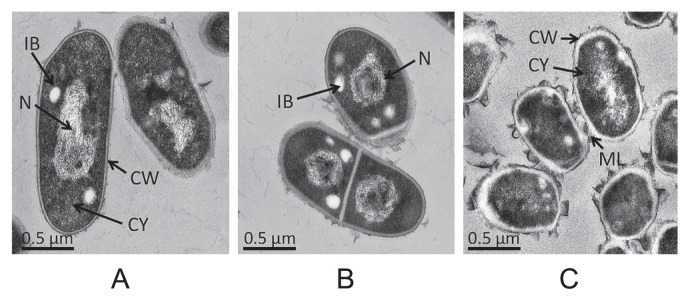
Morphological differences of *Tomitella biformata* cells at different growth phases. Representative transmission electron micrographs taken of (A) Early logarithmic phase (TSBF medium, 48 h); (B) Early stationary phase (TSBF medium, 72 h); (C) Oxygen-induced non-dividing cells (mMMF medium, oxygen-limited condition, 60 d). IB: inclusion body, CY: cytoplasm, N: nucleoid, CW: cell wall, ML: mucosal layer.

Non-dividing cells of *T. biformata* were induced under oxygen-limited culture conditions. After 60 d of incubation approximately 99% of the cells had lost cultivability on agar medium but retained membrane integrity (Fig. S3). TEM revealed that the majority of these cells had morphological characteristics distinct from cells in logarithmic and stationary growth phases (Fig. 5). Cells differed in shape, size, cell wall thickness, cell surface architecture and nucleoid density; features similar to those observed in other studies (2, 29). Not observed in *T. biformata* non-dividing cells were large electron transparent inclusions that were reported in *Mycobacterium smegmatis* non-dividing cells (21, 27).

### Resuscitation-promoting activity of rRpf fraction

**Fig. 6:**
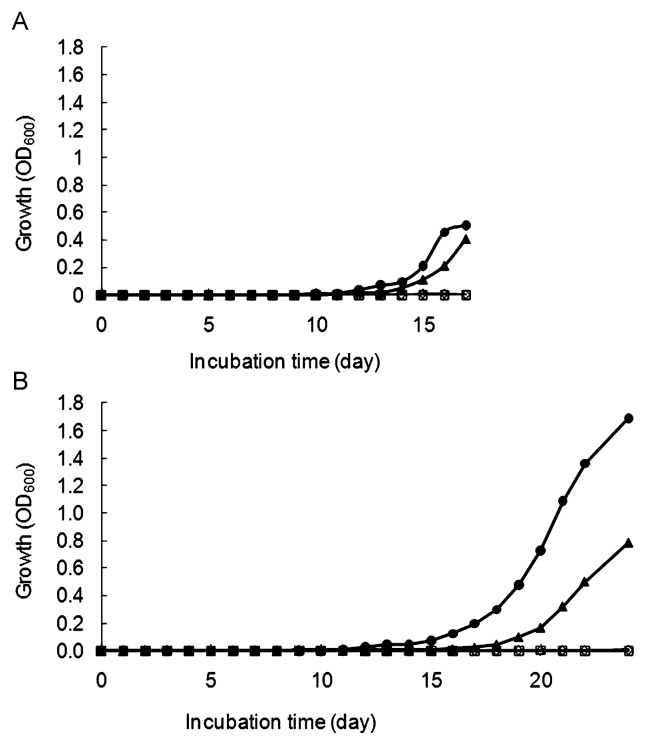
Two independent experiments using (A) 57.5 cells mL^−1^ and (B) 9.3 cell mL^−1^ of non-dividing cells (with no dividing cells present) demonstrating resuscitation of *Tomitella biformata* grown in liquid medium in the presence of rRpf. *T. biformata* cells from 60-d oxygen-limited cultures were washed and diluted prior to inoculation into mMMF medium containing final concentrations of 20 μg mL^−1^ (●) and 2 μg mL^−1^ of rRpf protein fraction (▲). Controls using sterilized distilled water (⋄), non-induced cell lysate fraction (□) (in A), and buffer from the purification step (□) (in B).

The rRpf fraction resuscitated *T. biformata* non-dividing cells obtained from 60-d oxygen-limited cultures (Fig. 6). Two independent experiments were performed using estimated initial cell numbers of 57.5 non-dividing cells mL^−1^ in the first experiment and a lower concentration of 9.3 non-dividing cells mL^−1^ in the second. Despite differences in initial cell concentrations, similar trends in resuscitation activity based on rRpf protein concentration were observed in both experiments and no increase in OD_600_ was seen in the negative control. Differences were only seen in the length of the lag phase. In the first experiment, OD_600_ started to increase after 12 and 14 d with the addition of 20 and 2 μg mL^−1^ rRpf protein, respectively (Fig. 6A). In the second experiment with a lower initial concentration of non-dividing cells (9.3 cells mL^−1^), increases in OD_600_ were observed at 15 and 19 d with the addition of 20 and 2 μg mL^−1^ rRpf protein, respectively (Fig. 6B); however, the nM concentration of rRpf *T. biformata* required to promote growth and resuscitation was much greater than the pM amounts of rRpf from *M. luteus* and *Myc. tuberculosis* reported in previous studies (17, 19). This difference in activity may stem from differences in the protein sequence and structure of *T. biformata* Rpf. This hypothesis warrants more in-depth genetic analysis in the future.

### Conclusion

As we initially hypothesized, a new *rpf* gene homolog was identified in *T. biformata*, a novel member of the *Actinobacteria* isolated from a unique environment. Phylo-genetic analysis of the deduced protein product indicated that *T. biformata* Rpf formed a monophyletic clade in the RpfB subfamily of Rpf and was distant from other functionally tested Rpfs. The whole length of the *rpf* gene from *T. biformata* had nucleotide sequence similarity to only one other bacterium, *Rhodococcus opacus* B4, in which Rpf has not been studied. The gene was successfully cloned and expressed to demonstrate that despite differences in sequences from previously studied Rpf, the protein was functional and could promote the growth of dividing cells in low-inoculum cultures and resuscitate non-dividing cells. This study provides additional genetic and functional information on the divergence of Rpf produced by different genera in *Actinobacteria*. It adds to the current body of knowledge of functional Rpf and will aid in future studies to understand other significant roles played by Rpf, such as intercellular communication among *Actinobacteria*.

## Supplementary Material
