# Supplementary material for: Resuscitation Promoting Factor (Rpf) from Tomitella biformata AHU 1821T Promotes Growth and Resuscitates Non-Dividing Cells
Source: Microbes Environ. 2012 Oct 26;28(1):58–64. doi: 10.1264/jsme2.ME12122 (PMC4070687; doi:10.1264/jsme2.ME12122)
Supplement: Supplementary file 1 [file 28_58_s1.pdf]

Table S1. [a]The putative number of *rpf* gene copies found in Actinobacteria finished and draft annotated genome sequences that are publically available.

[b]**Genome Name**	**Family**	[c]**Genome Size (bp)**	[d]***rpf* gene copies**	[e]**Habitat**	[f]**Accession no.**	**IMG id no.**	RefSeq Project ID	NCBI Taxon ID
Acidimicrobium ferrooxidans ICP, DSM 10331	Acidimicrobiaceae	2158157	1	Hot spring	NC_013124	644736322	57939	206672
Acidothermus cellulolyticus 11B	Acidothermaceae	2443540	1	Hot spring	NC_008578	639633001	57691	257309
Actinomyces coleocanis DSM 15436	Actinomycetaceae	1719346	1	Urogenital tract	Draft	643886017	58399	306537
Actinomyces odontolyticus ATCC 17982	Actinomycetaceae	2393758	1	Oral	Draft	640963058	58695	326424
Actinomyces odontolyticus F0309	Actinomycetaceae	2422895	1	Respiratory tract, oral	Draft	647000206	58397	106370
Actinomyces oris K20	Actinomycetaceae	2872429	1	Gastrointestinal tract	Draft	649989901	57759	281090
Actinomyces sp. oral taxon 170 str. F0386	Actinomycetaceae	3135160	1	Oral	Draft	651324005	57699	262316
Actinomyces sp. oral taxon 171 F0337	Actinomycetaceae	3002669	1	Oral	Draft	649989902	57695	233413
Actinomyces sp. oral taxon 178 F0338	Actinomycetaceae	2733433	0	Oral	Draft	649989903	57697	272631
Actinomyces sp. oral taxon 180 F0310	Actinomycetaceae	2348120	1	Oral	Draft	649989904	58465	164756
Actinomyces sp. oral taxon 448 str. F0400	Actinomycetaceae	2801370	0	Oral	Draft	651324006	57775	83331
Actinomyces sp. oral taxon 848 F0332	Actinomycetaceae	2518918	0	Oral	Draft	645951824	57777	83332
Actinomyces urogenitalis DSM 15434	Actinomycetaceae	2614023	1	Urogenital tract	Draft	643886015	58203	247156
Actinomyces viscosus C505	Actinomycetaceae	3133750	1	Respiratory tract	Draft	649989905	58101	267747
Actinosynnema mirum 101, DSM 43827	Actinosynnemataceae	8248144	3	Soil	NC_013093	644736323	58325	101510
Aeromicrobium marinum DSM 15272	Nocardioidaceae	2585197	0	Oral	Draft	645951844	58057	266117
Agreia sp. PHSC20C1	Microbacteriaceae	2769012	0	Sea water (surface)	Draft	638341246	57739	227882
Amycolatopsis mediterranei U32	Pseudonocardiaceae	10236715	2	Soil	NC_014318	648028006	57801	100226
Amycolicicoccus subflavus DQS3-9A1	Mycobacteriaceae	4863490	3	Crude oil-polluted soil	NC_015564	650716009	57703	269800
Arcanobacterium haemolyticum CCM, DSM 20595	Actinomycetaceae	1986154	0	Blood, pharyngeal mucosa	NC_014218	646564505	57961	218496
Arthrobacter arilaitensis re117, CIP108037	Micrococcaceae	3918192	2	Cheese	NC_014550	649633006	57705	203267
Arthrobacter aurescens TC1	Micrococcaceae	5226648	1	Atrazine contaminated soil	NC_008711	639633005	54109	321955
Arthrobacter chlorophenolicus A6	Micrococcaceae	4980870	2	Soil	NC_011886	643348509	54213	313589
Arthrobacter phenanthrenivorans Sphe3	Micrococcaceae	4535320	2	Creosote-contaminated soil	CP002379	650377905	54359	348776
Arthrobacter sp. FB24	Micrococcaceae	5070478	2	Chromate and xylene soil	NC_008541	639633006	54153	312284
Atopobium parvulum IPP 1246, DSM 20469	Coriobacteriaceae	1543805	0	Oral	NC_013203	644736327	61611	196627
Atopobium rimae ATCC 49626	Coriobacteriaceae	1626291	0	Oral	Draft	643886019	57905	196627

Atopobium vaginae DSM 15829	Coriobacteriaceae	1418601	0	Human vagina	Draft	648276620	58501	351607
Atopobium vaginae PB189-T1-4	Coriobacteriaceae	1449613	0	Human vagina	Draft	648276621	58141	290399
Beutenbergia cavernae HKI 0122, DSM 12333	Beutenbergiaceae	4669183	3	Soil (cave)	NC_012669	643692008	58559	367928
Bifidobacterium adolescentis ATCC 15703	Bifidobacteriaceae	2089645	0	Gastrointestinal tract	NC_008618	639633010	57693	243243
Bifidobacterium adolescentis L2-32	Bifidobacteriaceae	2385710	0	Gastrointestinal tract	Draft	640963015	58781	410289
Bifidobacterium angulatum DSM 20098	Bifidobacteriaceae	2000615	0	Gastrointestinal tract	Draft	642979361	57701	246196
Bifidobacterium animalis lactis AD011	Bifidobacteriaceae	1933695	0	Gastrointestinal tract	NC_011835	643348515	58491	189918
Bifidobacterium animalis lactis BB-12	Bifidobacteriaceae	1942198	0	Gastrointestinal tract	CP001853	646862308	58463	350058
Bifidobacterium animalis lactis Bl-04, ATCC SD5219	Bifidobacteriaceae	1938709	0	Gastrointestinal tract	NC_012814	644736329	58149	196162
Bifidobacterium animalis lactis DSM 10140	Bifidobacteriaceae	1938481	0	Gastrointestinal tract	CP001606	644736330	58489	164757
Bifidobacterium animalis lactis HN019	Bifidobacteriaceae	1915892	0	Gastrointestinal tract	Draft	641736235	62947	405948
Bifidobacterium animalis lactis V9	Bifidobacteriaceae	1944050	0	Gastrointestinal tract	CP001892	646862309	61625	443906
Bifidobacterium animalis subsp. lactis CNCM I-2494	Bifidobacteriaceae	1943113	0	Gastrointestinal tract	CP002915	651053004	58897	340322
Bifidobacterium bifidum NCIMB 41171	Bifidobacteriaceae	2186140	0	Gastrointestinal tract	Draft	643886040	59421	350054
Bifidobacterium bifidum PRL2010	Bifidobacteriaceae	2214656	0	Gastrointestinal tract	CP001840	649633015	58417	336982
Bifidobacterium bifidum S17	Bifidobacteriaceae	2186882	0	Gastrointestinal tract	CP002220	649633016	58853	419947
Bifidobacterium breve ACS-071-V-Sch8b	Bifidobacteriaceae	2327492	0	Human vagina	CP002743	651053005	58565	369723
Bifidobacterium breve DSM 20213	Bifidobacteriaceae	2297799	0	Gastrointestinal tract	Draft	643886102	54525	411903
Bifidobacterium breve UCC2003	Bifidobacteriaceae	2422684	0	Gastrointestinal tract	CP000303	651053006	58067	266940
Bifidobacterium catenulatum DSM 16992	Bifidobacteriaceae	2058429	0	Gastrointestinal tract	Draft	642979312	54549	411481
Bifidobacterium dentium ATCC 27678	Bifidobacteriaceae	2642081	0	Gastrointestinal tract	Draft	641736189	54529	411466
Bifidobacterium dentium ATCC 27679	Bifidobacteriaceae	2633776	0	Human vagina	Draft	648276623	58367	298653
Bifidobacterium dentium Bd1	Bifidobacteriaceae	2636367	0	Dental caries	CP001750	646311910	58899	288705
Bifidobacterium dentium JCVIHMP022	Bifidobacteriaceae	2636584	0	Human vagina	Draft	649989915	58659	391037
Bifidobacterium gallicum DSM 20093	Bifidobacteriaceae	2016380	0	Gastrointestinal tract	Draft	642979324	61639	504474
Bifidobacterium longum DJO10A	Bifidobacteriaceae	2389526	0	Gastrointestinal tract	CP000605	642555107	61613	36809
Bifidobacterium longum DJO10A	Bifidobacteriaceae	2375286	0	Gastrointestinal tract	Draft	645058795	59423	216594
Bifidobacterium longum infantis 157F-NC	Bifidobacteriaceae	2408831	0	Gastrointestinal tract	AP010890	649633017	54901	473819
Bifidobacterium longum infantis ATCC 15697	Bifidobacteriaceae	2832748	0	Gastrointestinal tract	CP001095	643348516	54453	395095
Bifidobacterium longum infantis JCM 1217	Bifidobacteriaceae	2385164	0	Gastrointestinal tract	AP010888	649633019	55085	486409
Bifidobacterium longum longum ATCC 55813	Bifidobacteriaceae	2372858	0	Gastrointestinal tract	Draft	643886140	54595	419947
Bifidobacterium longum longum BBMN68	Bifidobacteriaceae	2265943	0	Gastrointestinal tract	CP002286	649633018	58833	205913

Bifidobacterium longum longum CCUG 52486	Bifidobacteriaceae	2453376	0	Gastrointestinal tract	Draft	643886216	61577	31964
Bifidobacterium longum longum F8	Bifidobacteriaceae	2384987	0	Gastrointestinal tract	FP929034	650377912	59099	378753
Bifidobacterium longum longum JDM301	Bifidobacteriaceae	2477838	0	Gastrointestinal tract	CP002010	646564512	62939	362242
Bifidobacterium longum NCC2705	Bifidobacteriaceae	2260266	0	Gastrointestinal tract	AE014295	637000031	58983	455632
Bifidobacterium longum longum KACC 91563	Bifidobacteriaceae	2395764	0	Gastrointestinal tract	CP002794	651053008	55411	553204
Bifidobacterium pseudocatenulatum DSM 20438	Bifidobacteriaceae	2265534	0	Gastrointestinal tract	Draft	642979313	55097	515616
Bifidobacterium sp. 12_1_47BFAA	Bifidobacteriaceae	2401436	0	Gastrointestinal tract	Draft	649989916	55095	515615
Brachybacterium faecium 6-10, DSM 4810	Dermabacteraceae	3614992	**3**	Soil (poultry deep litter)	NC_013172	644736331	55099	515617
Brevibacterium linens BL2	Brevibacteriaceae	4366969	1	Cheese	Draft	638341022	55369	566552
Brevibacterium mcbrellneri ATCC 49030	Brevibacteriaceae	2561804	0	Urogenital tract	Draft	647000216	55303	547043
Catenulispora acidiphila ID139908, DSM 44928	Catenulisporaceae	10467782	**1**	Soil (forest)	NC_013131	644736339	55125	521003
Cellulomonas flavigena 134, DSM 20109	Cellulomonadaceae	4123179	**1**	Soil	NC_014151	646564520	54813	445975
Clavibacter michiganensis michiganensis NCPPB 382	Microbacteriaceae	3395237	0	Infected tomato	AM711867	640427108	55371	561180
Clavibacter michiganensis sepedonicus ATCC 33113	Microbacteriaceae	3403786	0	Infected potato	AM849034	642555124	55045	443255
Collinsella aerofaciens ATCC 25986	Coriobacteriaceae	2439869	0	Gastrointestinal tract	Draft	640612206	55273	537210
Collinsella intestinalis DSM 13280	Coriobacteriaceae	1789330	0	Gastrointestinal tract	Draft	642979320	55271	537209
Collinsella stercoris DSM 13279	Coriobacteriaceae	2399821	0	Gastrointestinal tract	Draft	642979321	55113	518635
Conexibacter woesei ID131577, DSM 14684	Conexibacteraceae	6359369	0	Soil (forest)	CP001854	646311917	55133	520140
Corynebacterium accolens ATCC 49725	Corynebacteriaceae	2413333	3	Respiratory tract	Draft	643886058	55131	520141
Corynebacterium accolens ATCC 49726	Corynebacteriaceae	2368466	3	Respiratory tract	Draft	648276632	58969	452863
Corynebacterium ammoniagenes DSM 20306	Corynebacteriaceae	2763612	2	Gastrointestinal tract	Draft	647000230	58911	442563
Corynebacterium amycolatum SK46	Corynebacteriaceae	2513912	3	Skin	Draft	642979306	58677	391904
Corynebacterium aurimucosum ATCC 700975	Corynebacteriaceae	2730325	2	Urogenital tract	Draft	645951870	59293	561304
Corynebacterium aurimucosum CN-1, ATCC 700975	Corynebacteriaceae	2819226	**2**	Urogenital tract	NC_012590	643692018	59047	471853
Corynebacterium bovis DSM 20582	Corynebacteriaceae	2522962	3	Soil	Draft	651324023	59409	169292
Corynebacterium diphtheriae gravis NCTC 13129	Corynebacteriaceae	2488635	**3**	Respiratory tract	NC_002935	637000082	59411	645127
Corynebacterium efficiens YS-314, DSM 44549	Corynebacteriaceae	3219505	2	Soil	NC_004369	644736345	59281	561275
Corynebacterium genitalium ATCC 33030	Corynebacteriaceae	2349653	3	Urogenital tract	Draft	648276633	55469	525263
Corynebacterium glucuronalyticum ATCC 51867	Corynebacteriaceae	2784713	2	Urogenital tract	Draft	643886147	55459	525246
Corynebacterium glucuronolyticum ATCC 51866	Corynebacteriaceae	2845674	2	Urogenital tract	Draft	643886137	55457	525245
Corynebacterium glutamicum Kalinowski ATCC 13032	Corynebacteriaceae	3282708	2	Soil	NC_012590	639279306	55409	553184
Corynebacterium glutamicum Nakagawa ATCC 13032	Corynebacteriaceae	3309401	**2**	Soil	NC_003450	639279307	55283	398513

Corynebacterium glutamicum R	Corynebacteriaceae	3363299	1	Soil	NC_009342	640427110	55471	525268
Corynebacterium jeikeium ATCC 43734	Corynebacteriaceae	2426461	3	Urogenital tract, blood	Draft	647000231	55467	525260
Corynebacterium jeikeium K411	Corynebacteriaceae	2476822	**3**	Skin	NC_007164	637000085	55463	525256
Corynebacterium kroppenstedtii DSM 44385	Corynebacteriaceae	2446804	**2**	Sputum	NC_012704	643692019	55395	525264
Corynebacterium lipophiloflavum DSM 44291	Corynebacteriaceae	2293743	2	Urogenital tract	Draft	643886002	55111	518634
Corynebacterium matruchotii ATCC 14266	Corynebacteriaceae	2855986	2	Oral	Draft	648276634	55889	585199
Corynebacterium matruchotii ATCC 33806	Corynebacteriaceae	2967145	2	Oral	Draft	643886207	55539	548478
Corynebacterium pseudogenitalium ATCC 33035	Corynebacteriaceae	2587776	3	Urogenital tract	Draft	643886084	55465	548480
Corynebacterium pseudotuberculosis 1002	Corynebacteriaceae	2335112	**2**	Lymph	CP001809	648231702	55397	548477
Corynebacterium pseudotuberculosis C231	Corynebacteriaceae	2328208	2	Lymph	CP001829	648231703	55853	596309
Corynebacterium pseudotuberculosis FRC41	Corynebacteriaceae	2337913	2	Lymph nodes	NC_007164	648028019	55379	566549
Corynebacterium pseudotuberculosis I19	Corynebacteriaceae	2337730	2	Lymph	NC_008268	650377927	55285	537937
Corynebacterium resistens DSM 45100	Corynebacteriaceae	2601311	3	Blood	NC_015673	650716029	59215	525909
Corynebacterium striatum ATCC 6940	Corynebacteriaceae	2724288	3	Urogenital tract	Draft	643886057	58951	446462
Corynebacterium tuberculostearicum SK141	Corynebacteriaceae	2372261	3	Skin	Draft	645058719	59195	521095
Corynebacterium ulcerans 809	Corynebacteriaceae	2502095	**2**	Lymph	CP002790	651053019	59359	580050
Corynebacterium ulcerans BR-AD22	Corynebacteriaceae	2606374	**3**	Lymph	NC_012522	650716030	59357	555970
Corynebacterium urealyticum DSM 7109	Corynebacteriaceae	2369219	3	Bladder, urinary tract	NC_010545	641522620	58649	446465
Corynebacterium variabile DSM 44702	Corynebacteriaceae	3299601	2	Soil	Draft	649989925	59077	479433
Cryptobacterium curtum 12-3, DSM 15641	Coriobacteriaceae	1617804	0	Oral	CP001682	644736346	62905	196164
Dermacoccus sp. Ellin185	Dermacoccaceae	3115364	2	Skin	Draft	649989926	59041	469378
Dietzia cinnamea P4	Dietziaceae	3555295	3	Oil-contaminated soil	Draft	649989929	59079	479437
Eggerthella lenta VPI 0255, DSM 2243	Coriobacteriaceae	3632260	0	Rectal tumor	CP001726	644736358	59053	471856
Eggerthella sp. 1_3_56FAA	Coriobacteriaceae	3388691	0	Gastrointestinal tract	Draft	649989930	59071	478801
Eggerthella sp. HGA1	Coriobacteriaceae	3362931	0	Gastrointestinal tract	Draft	651324028	59033	465515
Eggerthella sp. YY7918	Coriobacteriaceae	3123671	0	Gastrointestinal tract	AP012211	650716034	59069	478434
Frankia alni ACN14a	Frankiaceae	7497934	**2**	Soil (Plant root)	NC_008278	637000115	59221	479431
Frankia sp. CcI3	Frankiaceae	5433628	**2**	Soil (Plant root)	NC_007777	637000116	59055	471857
Frankia sp. EAN1pec	Frankiaceae	8982042	**2**	Soil (Plant root)	NC_009921	641228492	59051	471855
Frankia sp. EuI1c	Frankiaceae	8815781	2	Plant symbiont, soil	NC_014666	649633045	55399	555461
Frankia sp. EUN1f	Frankiaceae	9352740	2	Plant symbiont, soil	Draft	647000251	55413	553206
Frankia symbiont of Datisca glomerata	Frankiaceae	5204281	2	Plant symbiont, soil	Draft	647000253	55387	553481

Gardnerella vaginalis 317, ATCC 14019	Bifidobacteriaceae	1667350	0	Vaginal secretions	CP002104	649633046	55831	467200
Gardnerella vaginalis 409-05	Bifidobacteriaceae	1617544	0	Human vagina	CP001849	646311928	55007	487521
Gardnerella vaginalis 5-1	Bifidobacteriaceae	1672842	0	Vaginal secretions	Draft	648276677	54103	205913
Gardnerella vaginalis AMD	Bifidobacteriaceae	1606758	0	Vaginal secretions	Draft	647000255	55453	553201
Gardnerella vaginalis ATCC 14018	Bifidobacteriaceae	1604161	0	Human vagina	Draft	648276678	55545	457431
Gardnerella vaginalis HMP9231	Bifidobacteriaceae	1726517	0	Human vagina	CP002725	651053027	55547	457425
Geodermatophilus obscurus G-20, DSM 43160	Geodermatophilaceae	5322497	**1**	Desert soil	NC_013757	646311931	55543	566461
Gordonia bronchialis 3410, DSM 43247	Gordoniaceae	5290012	**5**	Sputum	NC_013441	646311932	55565	457430
Gordonia neofelifaecis NRRL B-59395	Gordoniaceae	4257286	3	Gastrointestinal tract	Draft	651324037	55385	557599
Gordonibacter pamelaeae 7-10-1-bT, DSM 19378	Coriobacteriaceae	3608022	0	Gastrointestinal tract	FP929047	650377943	55823	253839
Intrasporangium calvum 7KIP, DSM 43043	Intrasporangiaceae	4024382	**2**	Air	NC_014830	649633057	55829	591159
Janibacter sp. HTCC2649	Intrasporangiaceae	4228723	3	Sea water (10 m depth)	Draft	638341107	55825	457428
Jonesia denitrificans 55134, DSM 20603	Jonesiaceae	2749646	**1**	Blood	NC_013174	644736376	55827	457427
Kineococcus radiotolerans SRS30216	Kineosporiaceae	4956672	**2**	Radioactive polluted water	NC_009664	640753031	54945	478433
Kocuria rhizophila DC2201	Micrococcaceae	2697540	0	Typha (plant) rhizosphere	AP009152	642555133	54947	478435
Kribbella flavida IFO 14399, DSM 17836	Nocardioidaceae	7579488	**2**	Soil	NC_013729	646311938	55821	591158
Kytococcus sedentarius 541, DSM 20547	Dermacoccaceae	2785024	**2**	Marine, skin	NC_013169	644736380	55345	465515
Leifsonia xyli xyli CTCB07	Microbacteriaceae	2584158	0	Plant pathogen	AE016822	637000149	55987	649743
Microbacterium testaceum StLB037	Microbacteriaceae	3982034	0	Potato leaf endophyte	AP012052	650716057	55877	611303
Micrococcus luteus Fleming NCTC 2665	Micrococcaceae	2501097	**1**	Soil	NC_012803	644736390	55993	649764
Micrococcus luteus NCTC 2665	Micrococcaceae	2320592	1	Soil	Draft	645951813	55881	196164
Micrococcus luteus SK58	Micrococcaceae	2622687	0	Skin	Draft	647000274	55873	585531
Microlunatus phosphovorus NM-1	Propionibacteriaceae	5683123	**2**	Activated sludge	NC_015635	650716058	55819	591157
Micromonospora aurantiaca ATCC 27029	Micromonosporaceae	7025559	**1**	Soil	NC_014391	648028042	55879	611304
Micromonospora carbonacea africana ATCC 39149	Micromonosporaceae	6819904	0	Soil (Plant root)	Draft	647533185	55875	611302
Micromonospora sp. L5	Micromonosporaceae	6962533	**1**	Soil (Plant root)	NC_014815	649633069	55473	548476
Mobiluncus curtisii ATCC 43063	Actinomycetaceae	2146480	**1**	Vaginal secretions	NC_014246	648028043	43091	401473
Mobiluncus curtisii ATCC 51333	Actinomycetaceae	2111654	1	Vaginal secretions	Draft	649989973	43467	469383
Mobiluncus curtisii curtisii ATCC 35241	Actinomycetaceae	2136873	1	Human vagina	Draft	648276689	43211	553190
Mobiluncus curtisii holmesii ATCC 35242	Actinomycetaceae	2087529	1	Human vagina	Draft	649989974	43725	526225
Mobiluncus mulieris 28-1	Actinomycetaceae	2452380	1	Human vagina	Draft	647000277	41403	526226
Mobiluncus mulieris ATCC 35239	Actinomycetaceae	2464242	1	Human vagina	Draft	648276690	43465	479435

Mobiluncus mulieris ATCC 35243	Actinomycetaceae	2398290	1	Human vagina	Draft	643886108	43093	680646
Mobiluncus mulieris FB024-16	Actinomycetaceae	2384880	1	Human vagina	Draft	648276691	42521	479432
Mycobacterium abscessus CIP 104536	Mycobacteriaceae	5090491	4	Skin, Respiratory tract	NC_010397	641522641	41885	471852
Mycobacterium africanum GM041182	Mycobacteriaceae	4389314	5	Respiratory tract	NC_015758	650716059	41935	446471
Mycobacterium avium 104	Mycobacteriaceae	5475491	4	Respiratory tract	NC_008595	639633039	49489	644284
Mycobacterium avium avium ATCC 25291	Mycobacteriaceae	4857995	4	Liver, Respiratory tract	Draft	645058725	49131	759350
Mycobacterium avium paratuberculosis K-10	Mycobacteriaceae	4829781	4	Gastrointestinal tract	NC_002944	637000168	48821	446466
Mycobacterium bovis AF2122/97	Mycobacteriaceae	4345492	5	Respiratory tract	NC_002945	637000169	49483	446468
Mycobacterium bovis BCG Pasteur 1173P2	Mycobacteriaceae	4374522	5	Respiratory tract	NC_014830	639633040	48071	553199
Mycobacterium bovis BCG Tokyo 172	Mycobacteriaceae	4371711	4	Respiratory tract	NC_009664	643692028	13791	632772
Mycobacterium canettii CIPT 140010059	Mycobacteriaceae	4482059	4	Respiratory tract	NC_015848	650716060	40845	446469
Mycobacterium gilvum PYR-GCK	Mycobacteriaceae	5982829	4	River sediment	NC_013729	640427122	49049	640132
Mycobacterium gilvum Spyr1	Mycobacteriaceae	5783292	5	Creosote-contaminated soil	NC_014814	649633070	46663	446470
Mycobacterium intracellulare ATCC 13950	Mycobacteriaceae	5328562	4	Lymph nodes, lung	Draft	645058739	46531	680198
Mycobacterium kansasii ATCC 12478	Mycobacteriaceae	6400522	5	Respiratory tract	Draft	645058849	48999	469371
Mycobacterium leprae Br4923	Mycobacteriaceae	3268071	3	Skin	NC_011896	643348566	48829	521096
Mycobacterium leprae TN	Mycobacteriaceae	3268203	3	Skin	NC_002677	637000170	N/A	552531
Mycobacterium marinum M, ATCC BAA-535	Mycobacteriaceae	6660144	4	Respiratory tract	NC_010612	641522642	Draft	573236
Mycobacterium parascrofulaceum ATCC BAA-614	Mycobacteriaceae	6295508	5	Cervix biopsy	Draft	647000278	N/A	749414
Mycobacterium smegmatis MC2 155	Mycobacteriaceae	6988209	4	Soft tissue	NC_008596	639633041	46093	649742
Mycobacterium sp. JDM601	Mycobacteriaceae	4643668	5	Skin	NC_015576	650716061	48125	585530
Mycobacterium sp. JLS	Mycobacteriaceae	6048425	7	Creosote-contaminated soil	NC_002944	640069320	48813	649754
Mycobacterium sp. KMS	Mycobacteriaceae	6256079	6	Creosote-contaminated soil	NC_002945	639633042	40867	525262
Mycobacterium sp. MCS	Mycobacteriaceae	5920523	6	Creosote-contaminated soil	NC_008769	637000171	46069	102897
Mycobacterium tuberculosis 02_1987	Mycobacteriaceae	4443138	5	Respiratory tract	Draft	642979309	46257	656024
Mycobacterium tuberculosis 210	Mycobacteriaceae	4395332	5	Respiratory tract	Draft	647000279	49423	682147
Mycobacterium tuberculosis 94_M4241A	Mycobacteriaceae	4410654	5	Respiratory tract	Draft	642979310	46281	596312
Mycobacterium tuberculosis 98-R604 INH-RIF-EM	Mycobacteriaceae	4286999	5	Respiratory tract	Draft	645058721	42169	596328
Mycobacterium tuberculosis C	Mycobacteriaceae	4276200	5	Respiratory tract	Draft	638341130	48977	525368
Mycobacterium tuberculosis CCDC5079	Mycobacteriaceae	4398812	4	Respiratory tract	CP001641	651053043	42617	164513
Mycobacterium tuberculosis CCDC5180	Mycobacteriaceae	4405981	5	Respiratory tract	CP001642	651053044	47493	478433
Mycobacterium tuberculosis CDC1551	Mycobacteriaceae	4403706	5	Respiratory tract	NC_011896	637000172	47491	663887

Mycobacterium tuberculosis CPHL_A	Mycobacteriaceae	4376881	5	Respiratory tract	Draft	645951826	42957	679194
Mycobacterium tuberculosis EAS054	Mycobacteriaceae	4366920	5	Respiratory tract	Draft	642979363	46203	679195
Mycobacterium tuberculosis F11 (ExPEC)	Mycobacteriaceae	4424435	5	Respiratory tract	NC_008596	640427123	46201	686659
Mycobacterium tuberculosis GM 1503	Mycobacteriaceae	4261330	4	Respiratory tract	Draft	642979350	48621	525370
Mycobacterium tuberculosis H37Ra	Mycobacteriaceae	4419977	5	Respiratory tract	NC_009077	640427124	54491	405948
Mycobacterium tuberculosis H37Rv (lab strain)	Mycobacteriaceae	4411532	**5**	Respiratory tract	NC_000962	637000173	42963	647653
Mycobacterium tuberculosis Haarlem	Mycobacteriaceae	4347292	5	Respiratory tract	Draft	641736194	55817	219305
Mycobacterium tuberculosis K85	Mycobacteriaceae	4399672	5	Respiratory tract	Draft	645951854	47801	641144
Mycobacterium tuberculosis KZN 1435 (MDR)	Mycobacteriaceae	4398250	5	Respiratory tract	NC_012943	644736391	47829	641146
Mycobacterium tuberculosis KZN 4207	Mycobacteriaceae	4383975	5	Respiratory tract	Draft	647000280	47867	443255
Mycobacterium tuberculosis KZN 4207 (DS)	Mycobacteriaceae	4331915	4	Respiratory tract	Draft	645058860	48415	465543
Mycobacterium tuberculosis KZN 605 (XDR)	Mycobacteriaceae	4237019	5	Respiratory tract	Draft	645058861	47353	645465
Mycobacterium tuberculosis KZN R506	Mycobacteriaceae	4367618	5	Respiratory tract	Draft	648276692	50565	749927
Mycobacterium tuberculosis KZN V2475	Mycobacteriaceae	4356301	5	Respiratory tract	Draft	647000281	50585	765874
Mycobacterium tuberculosis SUMu001	Mycobacteriaceae	4356118	5	Respiratory tract	Draft	648276693	42501	644283
Mycobacterium tuberculosis SUMu002	Mycobacteriaceae	4321025	5	Respiratory tract	Draft	648276694	49695	548479
Mycobacterium tuberculosis SUMu003	Mycobacteriaceae	4345124	5	Respiratory tract	Draft	648276695	51367	633147
Mycobacterium tuberculosis SUMu004	Mycobacteriaceae	4338184	5	Respiratory tract	Draft	648276696	N/A	679896
Mycobacterium tuberculosis SUMu005	Mycobacteriaceae	4331659	5	Respiratory tract	Draft	648276697	N/A	681645
Mycobacterium tuberculosis SUMu006	Mycobacteriaceae	4328768	5	Respiratory tract	Draft	648276698	53885	525256
Mycobacterium tuberculosis SUMu007	Mycobacteriaceae	4321517	5	Respiratory tract	Draft	648276699	51527	866774
Mycobacterium tuberculosis SUMu008	Mycobacteriaceae	4327514	5	Respiratory tract	Draft	648276700	52349	871562
Mycobacterium tuberculosis SUMu009	Mycobacteriaceae	4320544	5	Respiratory tract	Draft	648276701	52361	862512
Mycobacterium tuberculosis SUMu010	Mycobacteriaceae	4352987	5	Respiratory tract	Draft	648276702	52785	585529
Mycobacterium tuberculosis SUMu011	Mycobacteriaceae	4340737	5	Respiratory tract	Draft	648276703	51885	553207
Mycobacterium tuberculosis SUMu012	Mycobacteriaceae	4325814	5	Respiratory tract	Draft	648276704	49673	682148
Mycobacterium tuberculosis T17	Mycobacteriaceae	4277414	5	Respiratory tract	Draft	642979349	53883	585528
Mycobacterium tuberculosis T46	Mycobacteriaceae	4347699	5	Respiratory tract	Draft	645951864	51715	585198
Mycobacterium tuberculosis T85	Mycobacteriaceae	4299331	4	Respiratory tract	Draft	642979364	52345	871571
Mycobacterium tuberculosis T92	Mycobacteriaceae	4260358	4	Respiratory tract	Draft	642979311	52965	866770
Mycobacterium ulcerans Agy99	Mycobacteriaceae	5805761	**4**	Skin, soil	NC_008611	642555140	47489	663886
Mycobacterium vanbaalenii PYR-1	Mycobacteriaceae	6491865	**5**	Oil-contaminated sediment	NC_008726	639633044	51927	675512

Nakamurella multipartita Y-104, DSM 44233	Nakamurellaceae	6060298	4	Active sludge	NC_013235	644736393	51925	675513
Nocardia farcinica IFM 10152	Nocardiaceae	6292344	4	Human lung	NC_006361	637000198	51931	675514
Nocardioidaceae bacterium Broad-1	Nocardioidaceae	5892013	1	-	Draft	651324077	51933	675515
Nocardioides sp. JS614	Nocardioidaceae	5293685	1	Soil	NC_008699	639633046	51935	675516
Nocardiopsis dassonvillei dassonvillei DSM 43111	Nocardiopsaceae	6543312	0	Blood	NC_014211	646564557	51937	675517
Olsenella uli VPI, DSM 7084	Coriobacteriaceae	2051896	0	Oral	NC_014363	648028047	51939	675518
Parascardovia denticolens DSM 10105	Bifidobacteriaceae	1891248	0	Oral	Draft	649989980	51941	675519
Parascardovia denticolens F0305	Bifidobacteriaceae	1897118	0	Oral	Draft	647533193	51943	675520
Propionibacterium acnes 266	Propionibacteriaceae	2494578	0	Pleuropulmonary infection	CP002409	651053058	51945	675521
Propionibacterium acnes 6609	Propionibacteriaceae	2560282	0	Skin	CP002815	651053059	51947	675522
Propionibacterium acnes J139	Propionibacteriaceae	2481963	0	Skin	Draft	647000296	51949	675523
Propionibacterium acnes J165	Propionibacteriaceae	2500083	0	Skin	Draft	647000297	52609	653045
Propionibacterium acnes KPA171202	Propionibacteriaceae	2560265	0	Skin	AE017283	637000215	50557	563032
Propionibacterium acnes SK137	Propionibacteriaceae	2495334	0	Skin	CP001977	646564561	59511	457429
Propionibacterium acnes SK187	Propionibacteriaceae	2510934	0	Skin	Draft	647000298	59513	463191
Propionibacterium freudenreichii shermanii CIRM-BIA1	Propionibacteriaceae	2616384	2	Cheese	NC_014215	649633084	53509	861360
Propionibacterium sp. 409-HC1	Propionibacteriaceae	2528533	0	Human vagina	Draft	651324091	59883	702459
Propionibacterium sp. 434-HC2	Propionibacteriaceae	2568635	0	Human vagina	Draft	651324092	59545	883062
Pseudonocardia dioxanivorans CB1190	Pseudonocardiaceae	7440794	5	Fresh water, sludge	CP002593	651053061	62693	565040
Pseudonocardia sp. P1	Pseudonocardiaceae	6388771	7	Ants symbiont	Draft	649989992	60163	890402
Renibacterium salmoninarum ATCC 33209	Micrococcaceae	3155250	2	Salmon kidney	NC_010168	641228502	62695	565042
Rhodococcus equi 103S	Nocardiaceae	5043170	7	Lung	NC_014659	649633089	42615	298654
Rhodococcus equi ATCC 33707	Nocardiaceae	5229298	7	Skin	Draft	647000305	55487	525284
Rhodococcus erythropolis PR4	Nocardiaceae	6895538	7	Sea water	NC_012490	643692033	61729	710696
Rhodococcus erythropolis SK121	Nocardiaceae	6785398	7	Wound, Skin	Draft	643886201	45895	648999
Rhodococcus jostii RHA1	Nocardiaceae	9702737	7	Soil	NC_008268	637000234	61403	278137
Rhodococcus opacus B4	Nocardiaceae	8834939	6	Soil?	NC_012522	646564564	49535	754252
Rothia dentocariosa ATCC 17931	Micrococcaceae	2506025	1	Oral	NC_014643	649633093	60171	685727
Rothia dentocariosa M567	Micrococcaceae	2532787	1	Oral, respiratory tract	Draft	648861015	49331	762948
Rothia mucilaginosa ATCC 25296	Micrococcaceae	2255158	1	Respiratory tract	Draft	645058800	63275	871541
Rothia mucilaginosa DY-18	Micrococcaceae	2264603	1	Respiratory tract	NC_013715	646311951	62285	706439
Rubrobacter xylanophilus DSM 9941	Rubrobacteraceae	3225748	0	Thermally polluted runoff	CP000386	637000248	62223	888051

Saccharomonospora viridis P101, DSM 43017	Pseudonocardiaceae	4308349	2	Lung	NC_013159	644736404	61455	888052
Saccharopolyspora erythraea NRRL 2338	Pseudonocardiaceae	8079083	4	Soil	Draft	647000310	51663	562973
Saccharopolyspora erythraea NRRL 2338 white	Pseudonocardiaceae	8212805	**4**	Soil	NC_009142	640069329	59471	553191
Salinispora arenicola CNS-205	Micromonosporaceae	5786361	3	Beach sand	CP000850	641228504	61873	469594
Salinispora tropica CNB-440	Micromonosporaceae	5183331	0	Coarse beach sand	CP000667	640427140	62003	858619
Sanguibacter keddieii ST-74, DSM 10542	Sanguibacteraceae	4253413	0	Bovine Blood	CP001819	646564565	59483	188626
Scardovia inopinata F0304	Bifidobacteriaceae	1804013	0	Dental caries	Draft	647533207	62173	910954
Segniliparus rotundus CDC 1076, DSM 44985	Segniliparaceae	3157527	**3**	Respiratory tract	NC_014168	646564566	61877	665943
Segniliparus rugosus ATCC BAA-974	Segniliparaceae	3567567	2	Respiratory tract	Draft	649989995	61495	887326
Slackia exigua ATCC 700122	Coriobacteriaceae	2094589	0	Deciduous teeth	Draft	645951837	61497	887899
Slackia heliotrinireducens RHS 1, DSM 20476	Coriobacteriaceae	3165038	0	Sheep rumen	CP001684	644736405	61281	864564
Stackebrandtia nassauensis LLR-40K-21, DSM44728	Glycomycetaceae	6841557	0	Soil	CP001778	646564571	63251	761194
Streptomyces albus J1074	Streptomycetaceae	6619469	4	Soil	Draft	645058823	61889	679197
Streptomyces avermitilis MA-4680	Streptomycetaceae	9119895	**5**	Soil	NC_003155	637000304	61899	683219
Streptomyces bingchenggensis BCW-1	Streptomycetaceae	11936683	**5**	Soil	CP002047	646862346	N/A	930171
Streptomyces clavuligerus ATCC 27064	Streptomycetaceae	6729086	0	Soil	Draft	642979328	N/A	657308
Streptomyces clavuligerus ATCC 27064	Streptomycetaceae	8556892	0	Soil	Draft	647533233	N/A	889513
Streptomyces clavuligerus ATCC 27064	Streptomycetaceae	9134976	5	Soil	Draft	651324105	N/A	722911
Streptomyces coelicolor A3(2)	Streptomycetaceae	9054847	**4**	Soil	NC_003888	637000305	67253	443218
Streptomyces ghanaensis ATCC 14672	Streptomycetaceae	8223278	5	Soil	Draft	645058824	50555	662755
Streptomyces griseoaurantiacus M045	Streptomycetaceae	7712377	5	Marine sediment	Draft	651324106	68291	945712
Streptomyces griseoflavus Tu4000	Streptomycetaceae	7364052	4	Soil	Draft	645058728	68707	502558
Streptomyces griseus griseus NBRC 13350	Streptomycetaceae	8545929	**3**	Soil	NC_010572	641522653	68055	1032480
Streptomyces griseus XyelbKG-1 1	Streptomycetaceae	8566464	3	Soil, host-associated	Draft	647000328	62789	979556
Streptomyces hygroscopicus ATCC 53653	Streptomycetaceae	10466286	4	Soil	Draft	645058857	68839	572418
Streptomyces lividans TK24	Streptomycetaceae	8190887	3	Soil	Draft	645058856	70731	78331
Streptomyces pristinaespiralis ATCC 25486	Streptomycetaceae	8133379	2	Soil	Draft	648861016	67369	875328
Streptomyces roseosporus NRRL 11379	Streptomycetaceae	7763119	3	Soil	Draft	645058827	66297	263358
Streptomyces roseosporus NRRL 15998	Streptomycetaceae	7560086	**3**	Soil	Draft	645058822	N/A	1042403
Streptomyces scabiei 87.22	Streptomycetaceae	10148695	4	Soil	NC_013929	646564576	N/A	866777
Streptomyces sp. AA4	Pseudonocardiaceae	9152172	4	Soil	Draft	645951800	N/A	326426
Streptomyces sp. C	Streptomycetaceae	7916041	4	Soil	Draft	645058853	N/A	391904

Streptomyces sp. e14	Streptomycetaceae	7928946	4	Soil	Draft	647533235	N/A	1031709
Streptomyces sp. Mg1	Streptomycetaceae	7105723	6	Soil	Draft	642791623	N/A	1035817
Streptomyces sp. SA3_actG	Streptomycetaceae	7443083	2	Soil	Draft	649990018	N/A	945711
Streptomyces sp. SirexAA-E	Streptomycetaceae	7372760	3	Soil	Draft	647000329	N/A	443150
Streptomyces sp. SPB74	Streptomycetaceae	6970553	3	Soil	Draft	647533234	N/A	1009464
Streptomyces sp. SPB78	Streptomycetaceae	6897976	3	Soil	Draft	645951849	N/A	443149
Streptomyces sp. Tu6071	Streptomycetaceae	7506727	3	Soil	Draft	651285011	N/A	909952
Streptomyces sviceus ATCC 29083	Streptomycetaceae	9313494	4	Soil	Draft	648861017	65087	675635
Streptomyces violaceusniger Tu 4113	Streptomycetaceae	10988130	5	Soil	Draft	648276750	66919	355249
Streptomyces viridochromogenes DSM 40736	Streptomycetaceae	8548109	5	Soil	Draft	645058855	66153	762963
Streptosporangium roseum NI 9100, DSM 43021	Streptosporangiaceae	10369518	0	Garden Soil	CP001814	646311958	70615	888056
Thermobifida fusca YX	Nocardiopsaceae	3642249	0	Compost, soil	CP000088	637000319	67345	927655
Thermobispora bispora R51, DSM 43833	Pseudonocardiaceae	4189976	0	Decaying manure	CP001874	646564581	63585	910311
Thermomonospora curvata DSM 43183	Thermomonosporaceae	5639016	0	Compost	CP001738	646311963	64473	644548
Tropheryma whipplei TW08/27	Microbacteriaceae	925938	0	Cerebrospinal fluid	BX072543	637000330	64407	408672
Tropheryma whipplei Twist	Microbacteriaceae	927303	0	Cardiac valve	AE014184	637000331	67825	936047
Tsukamurella paurometabola 33, DSM 20162	Tsukamurellaceae	4479724	**3**	Host-associated, sludge, soil	NC_014158	646564587	67827	936048
Verrucosispora maris AB-18-032	Micromonosporaceae	6732271	0	Deep marine sediment	CP002638	650716103	64411	443255
Xylanimonas cellulosilytica XIL07, DSM 15894	Promicromonosporaceae	3831380	0	Decaying tree	CP001821	646311968	66149	996637

[a] This table was modified from the table first published by Ravagnani et al. (2005) by the addition of recently available genome sequences and habitat metadata.

[b] The list includes all the Actinobacteria finished and draft annotated genome sequences available in the integrated microbial genomes (IMG) database (Markowitz et al., 2012). Genome information was downloaded from IMG.

[c] Estimated genome sizes (if the information was available).

[d] The number of Rpf sequences in each genome was found using a BLASTp search (Altschul et al., 1997) in IMG using the Rpf domain sequence from *Micrococcus luteus* and E-value of 1e-5. The putative number of *rpf* gene copies in each Actinobacteria genome is given but values for draft genomes

are tentative since annotations have not been completed. Numbers in blue were sequences used to make Supplemental Fig. S1. A search of all genomes in the IMG database using the same BLASTp search criteria did not identify any Rpf domains in genomes from other phyla.

[e] Available information about the habitat from which the bacteria were isolated.

[f] Accession numbers for draft sequences are not currently available.

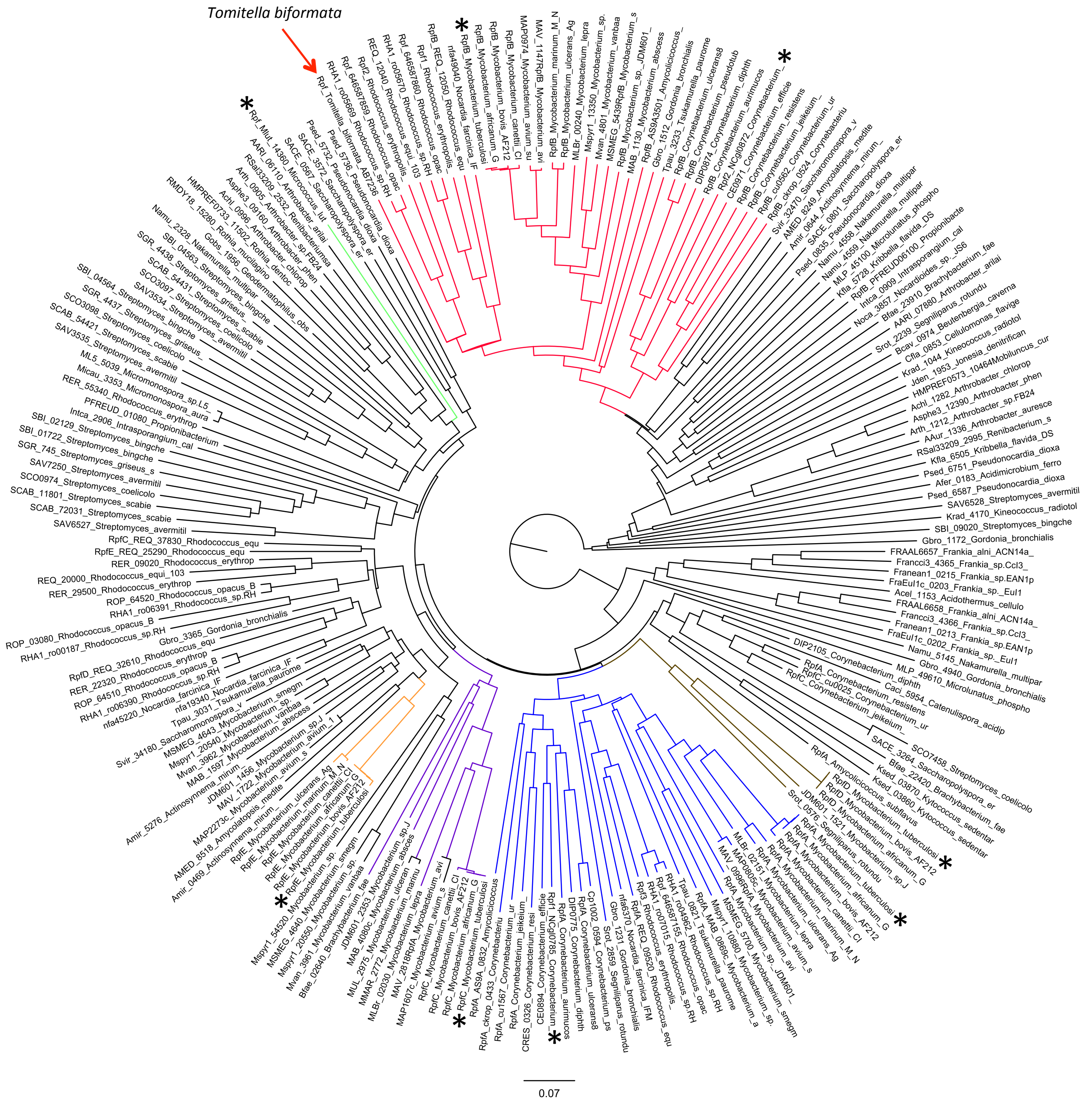

Fig. S1. Neighbor-joining tree of ClustalW2 aligned deduced amino acid sequence from *T. biformata rpf* gene and related proteins from completed genome sequences of Actinobacteria (see Table S1 for details). Potential Rpf subfamilies based on relatedness to *Mycobacterium tuberculosis* sequences in which functional analyses have been performed (Mukamolova et al., 2002), RpfA (blue branches), RpfB (red branches), RpfC (purple branches), RpfD (brown branches), RpfE (orange branches) and unclassified (black branches). Green branch is Rpf from *Micrococcus luteus* whose function has been tested and falls into the LysM subfamily according to the classification of Ravagnani et al. (2005). Asterisks indicate proteins whose functional activities have been tested.

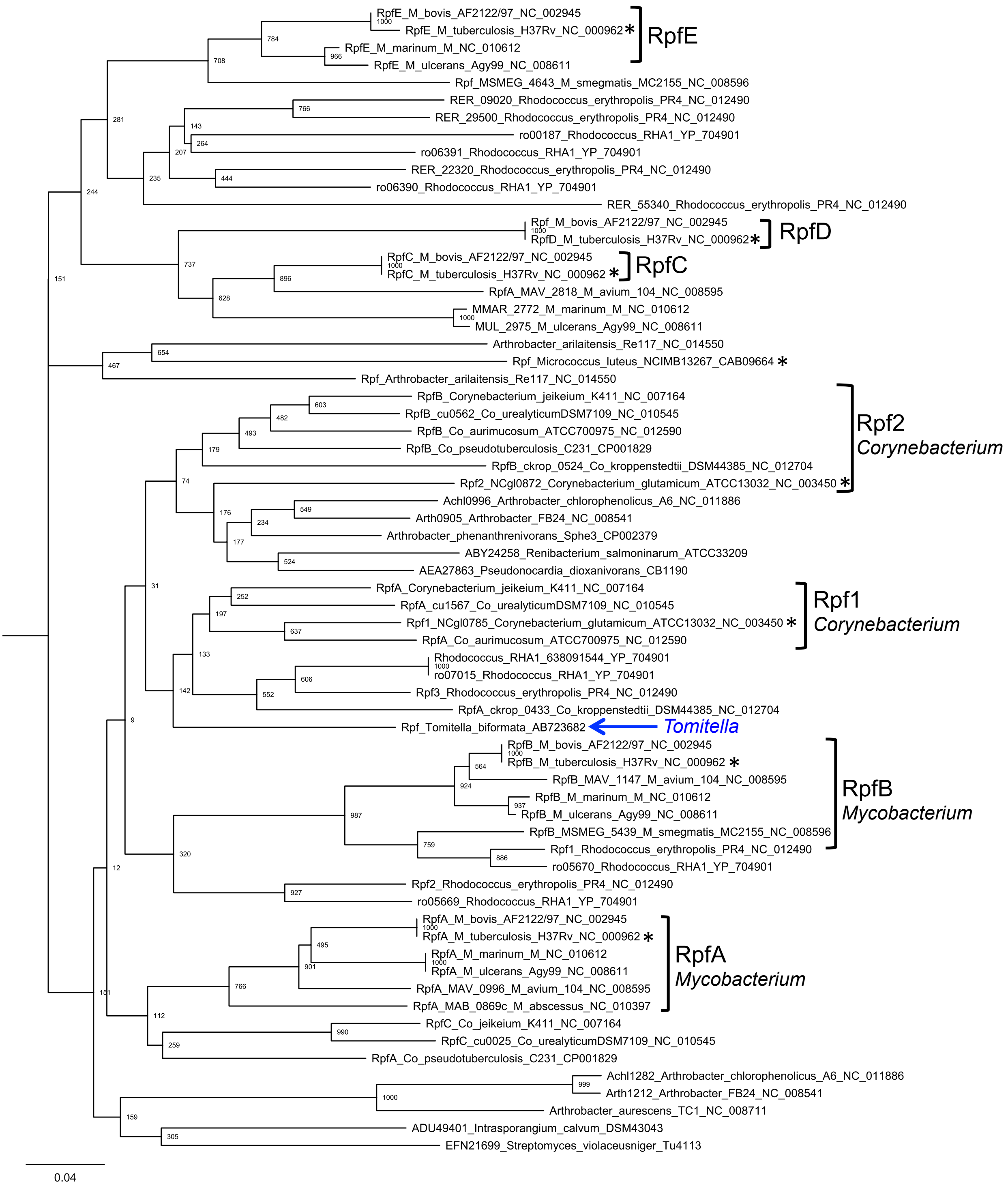

Fig. S2. Neighbor-joining tree of ClustalW2 aligned deduced amino acid sequence of the Rpf domain of *T. biformata rpf* gene and from select species from completed genome sequences of Actinobacteria. Clades of the five *Mycobacterium* and two *Corynebacterium* Rpf subfamilies are noted. Asterisks indicate proteins whose functional activities have been tested. Accession numbers of genomes are included in the tree.

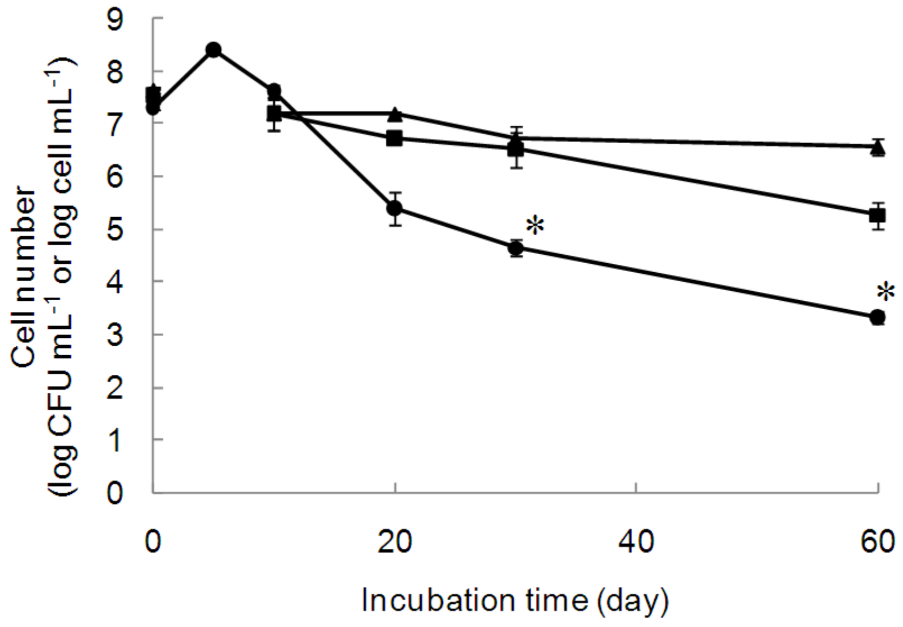

Fig. S3. Induction of *Tomitella biformata* cells into a non-dividing state by prolonged incubation in mMMF under oxygen limited conditions at 20°C. Number of total cells in population (▲), live cells determined by PI impermeability (■), and CFU count on TSBF agar medium (●). Bar depict standard deviations (n=3). *Significantly different ($p<0.05$) number of dividing cells compared to total number of live cells based on paired t-test.

References for Supplemental:

Altschul, S. F., T. L. Madden, A. A. Schäffer, J. Zhang, Z. Zhang, W. Miller, and D. J. Lipman. 1997. Gapped BLAST and PSI-BLAST: a new generation of protein database search programs. Nucleic Acids Research **25:**3389-3402.

Markowitz, V. M., I. M. A. Chen, K. Palaniappan, K. Chu, E. Szeto, Y. Grechkin, A. Ratner, B. Jacob, J. H. Huang, P. Williams, M. Huntemann, I. Anderson, K. Mavromatis, N. N. Ivanova, and N. C. Kyrpides. 2012. IMG: the integrated microbial genomes database and comparative analysis system. Nucleic Acids Research **40:**D115-D122.

Mukamolova, G. V., O. A. Turapov, D. I. Young, A. S. Kaprelyants, D. B. Kell, and M. Young. 2002. A family of autocrine growth factors in *Mycobacterium tuberculosis*. Mol. Microbiol. **46:**623-635.

Ravagnani, A., C. Finan, and M. Young. 2005. A novel firmicute protein family related to the actinobacterial resuscitation-promoting factors by non-orthologous domain displacement. BMC Genomics **6:**39-53.
